# Glutamine Supplementation and Exercise: A Narrative Review of Biochemical Mechanisms and Timing Strategies

**DOI:** 10.3390/medicina62020329

**Published:** 2026-02-06

**Authors:** Branka Djordjevic, Vladana Stojiljkovic, Aleksandra Velickov, Jana Kocic, Jelena Milenkovic, Andrej Veljkovic, Jelena Basic, Tatjana Cvetkovic

**Affiliations:** 1 Department of Biochemistry, Faculty of Medicine, University of Nis, Bulevar Dr. Zorana Djindjica 81, 18000 Nis, Serbia; vladana.stojiljkovic@medfak.ni.ac.rs (V.S.); jana.kocic@medfak.ni.ac.rs (J.K.); andrej.veljkovic@medfak.ni.ac.rs (A.V.); jelena.basic@medfak.ni.ac.rs (J.B.); tatjana.cvetkovic@medfak.ni.ac.rs (T.C.); 2 Department of Histology and Embryology, Faculty of Medicine, University of Nis, Bulevar Dr. Zorana Djindjica 81, 18000 Nis, Serbia; aleksandra.velickov@medfak.ni.ac.rs; 3 Department of Pathophysiology, Faculty of Medicine, University of Nis, Bulevar Dr. Zorana Djindjica 81, 18000 Nis, Serbia; jelena.milenkovic@medfak.ni.ac.rs

**Keywords:** glutamine, exercise, supplementation timing, muscle recovery, athletes

## Abstract

Intense physical activity imposes substantial oxidative, metabolic, and immunological stress on the human body. It is often accompanied by reductions in plasma glutamine levels, making this amino acid conditionally essential. Glutamine plays a vital role in energy production, nitrogen transport, acid–base balance, antioxidant defense, and immune function. It is required in the biosynthesis of neurotransmitters, nucleotides, nicotinamide-derived coenzymes, glutathione, and hexosamines, making it a candidate for supporting exercise recovery. In addition, glutamine may support key mechanisms involved in muscle adaptation and recovery during exercise-induced stress by contributing to redox balance, energy sensing, anabolic signaling, intestinal barrier integrity, and immune function. This narrative review aims to synthesize biochemical mechanisms underlying glutamine effects relevant to exercise and evaluate preclinical and clinical findings on supplementation outcomes, with emphasis on timing strategies. Preclinical findings demonstrate that glutamine can modulate protein synthesis, reduce oxidative stress, improve intestinal integrity, and attenuate immune and inflammatory disturbances. Limited preclinical data suggest that post-exercise supplementation may better resolve muscle and organ damage. Clinical trials, however, report heterogeneous outcomes: several studies show improvements in markers of intestinal permeability and intestinal epithelial damage, oxidative stress, muscle damage, and inflammation, whereas others report minimal or no effect, including limited influence on performance outcomes. Variability in timing protocols, participant characteristics, and measured endpoints contributes to inconsistent findings. Overall, glutamine demonstrates several biologically plausible mechanisms that could support recovery and overall health in active individuals, athletes, and specific clinical populations. However, current evidence remains insufficient to determine clear supplementation benefits or define an optimal timing strategy. Future research using standardized protocols and integrated biochemical and functional endpoints is needed to clarify timing effects. Until such evidence emerges, recommendations should remain individualized, considering athlete-specific needs.

## 1. Introduction

Intense physical activity (PA) places significant metabolic, oxidative, and immunological stress on the body, reflected in changes in cellular metabolite levels, increased leukocyte mobilization followed by cytokine release, and an increase in production of reactive oxygen species (ROS) [1,2]. Due to the potential to alleviate exercise-induced stress, nutrition and supplementation strategies have gained attention for optimizing recovery and performance in individuals who exercise. Among amino acids, glutamine stands out due to its involvement in nitrogen exchange between tissues, redox homeostasis, immune cell function, and maintaining intestinal mucosa integrity, all of which are essential during periods of intense exercise [3,4,5,6,7,8].

Apart from being an energy source, glutamine is a substrate or amino group donor for the various synthesis reactions in human cells, including production of other amino acids (glutamate, arginine, proline), nicotinamide-derived coenzymes, tripeptide glutathione, hexosamines, and nucleotides [5,9]. These biochemical pathways are crucial to energy production, antioxidant defense, glycosylation, and cell division. In the context of exercise-induced stress, they provide a rationale on how glutamine availability could affect muscle recovery, immune, and intestinal barrier function. Through glutaminolysis, it supplies carbon to the tricarboxylic acid (TCA) cycle, supporting energy production [10]. It contributes to ammonia detoxification through transamination reactions and enables renal synthesis of ammonia, which helps in acid–base balance regulation. As a precursor of glutathione, glutamine is essential for maintaining antioxidant defenses and limiting ROS-induced cellular damage [8].

In the immune system, glutamine serves as a key substrate for lymphocytes and macrophages, supporting not only energy production but also nucleotide synthesis required for cell proliferation [5]. In addition, it influences cytokine release, thereby affecting both the magnitude and resolution of exercise-induced inflammation [4]. In the intestine, it provides energy for enterocytes and helps in preserving tight-junction integrity, which might be disrupted during intense exercise [3,7]. Furthermore, its involvement in nicotinamide-derived coenzyme biosynthesis and the hexosamine pathway links glutamine availability to redox balance, protein O-GlcNAcylation, and cellular stress signaling, which are relevant to metabolic regulation and muscle repair after strenuous exercise [11,12,13,14].

Normally, glutamine is synthesized in sufficient amounts in the body; however, its level can become depleted during prolonged or high-intensity exercise, overtraining, trauma, or illness [15,16,17,18]. Under such conditions, glutamine becomes deficient and is considered conditionally essential [3,5,19]. Exercise-induced reduction in plasma glutamine levels can be corrected by glutamine supplementation, which may help in maintaining metabolic processes during exercise or supporting recovery post-exercise [4,20]. However, the practical significance of glutamine level correction by supplementation remains a topic of active investigation, and currently, there is no recommendation for routine supplementation in individuals who exercise.

Glutamine supplementation is demonstrated to be effective in reducing muscle soreness, accelerating recovery, and supporting protein synthesis in animal studies [21,22]. In addition, recent pre-clinical studies suggest that the timing of supplementation, whether before or after exercise, may modulate its outcomes [21]. Clinical studies, however, report mixed results with some studies suggesting beneficial effects on oxidative stress, inflammation, intestinal mucosa integrity, and muscle damage biomarkers, while others show limited or no effects [23,24,25,26,27,28]. The discrepancies in outcomes might be due to differences in dosing, glutamine formulation, timing protocols, participant training status, and type of exercise.

A recent meta-analysis, which included studies published before January 2017, found no significant effects on immune cell counts, aerobic capacity, or body composition, but noted reductions in body weight and neutrophil counts at higher doses and increased post-exercise blood glucose with dipeptide formulations [29]. Whether the timing of glutamine supplementation exerts a meaningful influence on its effects and efficacy remains unclear. Previous reviews have addressed glutamine supplementation outcomes, yet rarely assessed the biochemical basis for timing effects or integrated findings from both clinical and preclinical research.

This narrative review aims (i) to synthesize the biochemical and physiological mechanisms that support glutamine relevance to exercise and (ii) to evaluate preclinical and clinical evidence for outcomes of glutamine supplementation in exercising individuals with emphasis on the role of timing, whether pre-, post-exercise, or combined administration protocol. Given the diversity of published studies, we also included investigations in exercising individuals from specific clinical populations when these trials reported relevant biochemical effects related to glutamine supplementation.

## 2. Materials and Methods

A comprehensive literature search was conducted in PubMed and MEDLINE to identify studies evaluating glutamine supplementation in relation to exercise, covering publications in English from September 2015 until September 2025. Databases were searched using the following terms: (“glutamine” [MeSH] OR “glutamine supplementation” [Title/Abstract]) AND (“exercise” [MeSH] OR “physical activity” [Title/Abstract] OR “training” [Title/Abstract] OR “sports” [MeSH] OR “athletic performance” [MeSH] OR “athletes” [Title/Abstract]). To synthesize the biochemical mechanisms that support glutamine relevance to exercise, we conducted multiple additional targeted searches in PubMed using keywords related to glutamine metabolism, exercise-induced stress, and biochemical pathways to identify relevant papers. The initial search was conducted by B.Dj. and V.S. in September 2025, and additional targeted searches were performed by all authors until December 2025. Screening of records (titles/abstracts and full texts) was conducted by B.Dj. and V.S. All steps were performed manually; no screening software was used.

Initial search found 84 articles (including 17 reviews) which were screened for eligibility by screening titles and abstracts against predefined inclusion criteria. We included original preclinical (rodent) and clinical studies that evaluated oral glutamine (free or dipeptide) in the context of exercise. We excluded studies on parenteral nutrition, critically ill patients, and studies unrelated to exercise. For clinical studies, randomized designs were prioritized. We categorized timing as pre-exercise, post-exercise, combined (pre+post), or unspecified. Although this is a narrative review, we documented basic quality domains (randomization/blinding, sample size). However, no formal risk-of-bias or quality assessment was performed. After full-text screening, 23 clinical trials (predominantly randomized controlled trials) and 10 preclinical studies were included. Data extracted from eligible studies included population characteristics, supplementation protocol (form, dose, duration, timing), study design, exercise protocol, and main findings.

## 3. Glutamine Biochemistry

Glutamine is, along with alanine, the most abundant free amino acid in human plasma. Concentration of 0.5–0.7 mmol/L accounts for up to one third of the total free amino acid pool [30]. High circulating concentration of glutamine in plasma is a probable consequence of its prominent role in inter-organ nitrogen transport. The major glutamine-producing organs are the brain, skeletal muscles, adipose tissue, and liver [9]. However, due to their size and metabolic roles, skeletal muscles are considered the largest and most important glutamine storage sites in the human body [31].

The primary consumers of glutamine are the kidneys due to their function in acid-base balance and gluconeogenesis, and rapidly dividing cells such as enterocytes and leukocytes [9,30]. Requirements for glutamine in rapidly dividing cells are high because glutamine simultaneously represents a source of nitrogen for the purine and pyrimidine synthesis and an energy source through the process of glutaminolysis [32]. Additionally, glutamine contributes to the synthesis of aspartate and ornithine, supporting nucleotide synthesis and the urea cycle, respectively.

In catabolic conditions such as overtraining or recovering from major trauma or surgery, glutamine release from the muscles is decreased due to reduced stores, muscle inactivity, suppressed synthesis, and transmembrane transport [30,33]. Decreased production and excessive demand lead to a reduction in plasma glutamine concentration to up to 50%, which is associated with poor prognosis in critically ill patients [9,16,30,34]. Animal studies show that resistance exercise reduces glutamine concentration in plasma and muscles, whereas supplementation suppresses the decline [4,8,35].

Multiple membrane transporters with distinct transport mechanisms maintain glutamine homeostasis by regulating its absorption, reabsorption, and distribution to tissues. Cellular glutamine uptake is mediated primarily by transporters belonging to solute carrier (SLC) 1, 6, 7, and 38 families [36]. Transporters like SLC1A5/ASCT2, SLC38A1/SNAT1, and SLC38A2/SNAT2 have a high affinity for glutamine but also transport other neutral amino acids in the cell using a Na^+^-dependent mechanism [36,37]. While those transporters mediate glutamine uptake in most cells, apical SLC6A19/B0AT1 contributes to glutamine uptake in epithelial cells, including the small intestine [36]. In contrast, SLC7A5/LAT1 is an obligatory antiporter for large neutral amino acids that uses intracellular glutamine as the exchange substrate [38]. Since most of the essential amino acids, including leucine, belong to the large neutral amino acids category, this transport mechanism represents a link between glutamine pools to leucine influx and mammalian (or mechanistic) target of rapamycin (mTOR) activation [38,39].

### 3.1. Glutamate–Glutamine Cycle and Glutaminolysis

The biochemical conversion of glutamine to glutamate and vice versa is called the glutamate–glutamine cycle. In the initial step of glutaminolysis, glutamine generates glutamate and ammonia, thereby supporting energy production and biosynthetic processes in rapidly dividing cells such as enterocytes, leukocytes, and cancer cells [10,40]. Ammonia produced in this reaction supports the urea cycle in the liver, while the same reaction represents a source of ammonium ions that are excreted in the urine, contributing to the acid-base balance.

**Figure 1:**
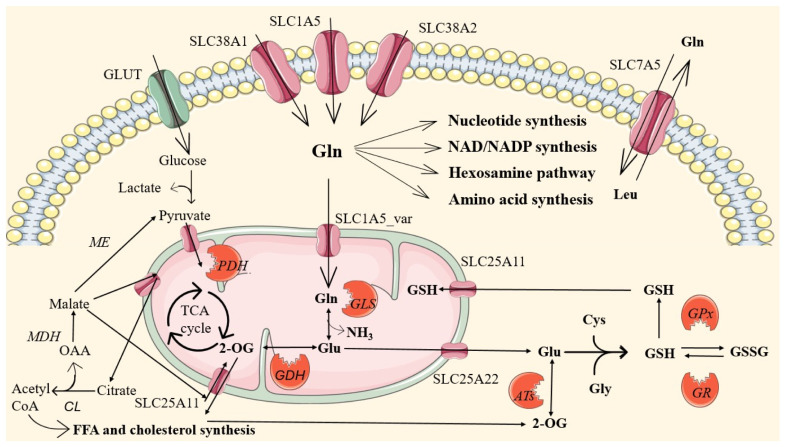
Cellular transport and metabolic pathways of glutamine (Gln) and related molecules. Glutamine enters the cell via solute carrier transporters (SLC38A1, SLC1A5, SLC38A2) and participates in multiple biosynthetic and energy-related pathways (glutaminolysis). Abbreviations: 2-OG—2-oxoglutarate; CL—citrate lyase; Cys—cysteine; GDH—glutamate dehydrogenase; GLS—glutaminase; GLUT—glucose transporter; Glu—glutamate; Gly—glycine; Gln—glutamine; GPx—glutathione peroxidase; GR—glutathione reductase; GSH—reduced glutathione; GSSG—oxidized glutathione; MD—malate dehydrogenase; ME—malic enzyme; NAD/NADP—nicotinamide adenine dinucleotide/nicotinamide adenine dinucleotide phosphate; OAA—oxaloacetate; PDH—pyruvate dehydrogenase; SLC—solute carrier transporter; TCA—tricarboxylic acid.

During glutaminolysis, glutamate is further converted to 2-oxoglutarate by glutamate dehydrogenase, which then enters the tricarboxylic acid (TCA) cycle to form malate and oxaloacetate [10]. In the TCA cycle, oxaloacetate is further converted to citrate, which might be exported out of mitochondria and used to feed cytosolic pools of oxaloacetate and malate. In the cytosol, malate can be further converted to pyruvate by malic enzyme, providing substrate for anaerobic glycolysis or acetyl-CoA, which contributes to fatty acid synthesis [32]. Additionally, through transamination reactions, pyruvate and oxaloacetate can be converted to alanine and aspartate, respectively [10] (Figure 1).

In the reaction of glutamine synthesis, glutamate is converted back to glutamine via the adenosine triphosphate (ATP)-dependent enzyme glutamine synthetase, thereby removing one molecule of ammonia. This reaction is crucial for detoxifying ammonia and maintaining glutamine pools during catabolic stress events such as exhaustive exercise, infection, or trauma. Glutamine synthetase is therefore highly expressed in the liver, the muscles, and the brain [31,41]. In the brain, glutamine metabolism produces glutamate and γ-aminobutyric acid (GABA), which act as excitatory and inhibitory neurotransmitters, respectively [9]. Apart from sustaining neurotransmitter synthesis, glutamate is central to energy metabolism and ammonia detoxification [42,43,44]. Dysregulation in the glutamate–glutamine cycle in the brain of animals exposed to strenuous exercise might contribute to the exercise-related central fatigue phenomenon [45].

### 3.2. Glutamine and the Synthesis of Nicotinamide-Derived Coenzymes

Beyond its role in energy metabolism, glutamine plays a crucial role as an amino group donor in various biosynthetic reactions, including the synthesis of nucleotides, nicotinamide adenine dinucleotide (NAD+), and nicotinamide adenine dinucleotide phosphate (NADP+), hexosamines, and glutathione. Glutamine is needed in the last step of the NAD+ synthesis, while NADP+ is generated by subsequent phosphorylation of NAD+. The reaction of NAD synthesis is ATP-dependent enzymatic amidation of deamido-NAD+ catalyzed by NAD+ synthetase [9,14]. Deamido-NAD+ can be generated de novo from tryptophan or via salvage pathways from dietary niacin.

NAD+ and NADP+ are essential coenzymes involved in enzymatic redox reactions. NAD+/NADH are involved in energy production in glycolysis and the TCA cycle, while NADP+/NADPH supports the synthesis of fatty acids and cholesterol. NADPH also serves as a coenzyme for NADPH oxidase 2 (NOX2), which is responsible for the production of ROS and respiratory burst in leukocytes that enables the fight against microorganisms [46]. Apart from its role in the immune system, NADPH is required by the enzyme glutathione reductase to regenerate reduced glutathione, a key endogenous antioxidant that protects cells from oxidative stress [13].

### 3.3. Glutamine and Oxidative Stress

Oxidative stress represents an imbalance between the production of ROS and the capacity of the body’s antioxidant defenses. ROS are naturally produced during exercise, especially in skeletal muscle, due to increased oxygen consumption. Interestingly, moderate increases in ROS production during exercise contribute to skeletal muscle adaptation to endurance training [47]. However, excessive oxidative stress from strenuous exercise may lead to oxidative damage of macromolecules in muscle fibers, accelerated fatigue, and delayed recovery [2,47].

Glutamine supports anti-oxidative defense, directly by supplying glutamate for glutathione (GSH) synthesis and, indirectly, by NADPH-dependent GSH recycling through glutathione reductase. GSH is a tripeptide composed of glutamate, cysteine, and glycine (γ-L-glutamyl-L-cysteinyl-glycine), and functions as a major endogenous antioxidant in cells [13]. It serves as a cofactor for glutathione peroxidase, an enzyme that degrades hydrogen peroxide within cells. During this reaction, reduced GSH is oxidized and dimerizes, forming a disulfide bond between two GSH molecules to produce glutathione disulfide (GSSG) [13]. The oxidized form (GSSG) can be reduced back to GSH by the action of NADPH-dependent glutathione reductase.

During PA, elevated oxygen consumption increases ROS production, which disrupts the cellular redox state, including the ratio of reduced to oxidized glutathione (GSH/GSSG). Preclinical studies demonstrate that glutamine supplementation increases intracellular levels of GSH and restores redox balance [8]. Furthermore, animal studies show that while exercise reduces the levels of anti-oxidative enzymes, including glutathione peroxidase in muscle tissue, glutamine supplementation can restore them [48]. Beyond its antioxidant role, glutamine influences cellular stress responses through the hexosamine pathway and heat shock protein regulation [9,49].

### 3.4. Glutamine, Hexosamine Pathway, and Heat Shock Proteins

The hexosamine biosynthetic pathway (HBP) integrates glucose and glutamine metabolism with regulation of cellular signaling [11]. Glycolysis intermediate fructose-6-phosphate and glutamine form glucosamine-6-phosphate in a reaction catalyzed by an enzyme glutamine: fructose-6-phosphate amidotransferase (GFAT) [50]. The reaction product is subsequently converted to UDP-N-acetylglucosamine (UDP-GlcNAc). UDP-GlcNAc serves as a donor for O-GlcNAcylation, a reversible post-translational modification of serine/threonine residues on nuclear and cytoplasmic proteins. This modification influences key processes such as glucose and lipid metabolism, cell signaling, transcriptional regulation, cellular stress, and host–pathogen interactions [11,12].

Elevated flux through the HBP, often associated with high glucose and glutamine availability, can alter insulin sensitivity and metabolic homeostasis, linking this pathway to conditions such as diabetes and metabolic syndrome [50]. Under physiological conditions, O-GlcNAcylation contributes to lipid metabolism homeostasis to influence food intake, lipid utilization, storage, and release, suggesting a link with obesity and dyslipidemia [12]. In addition, HSP has been implicated in the regulation of immune cells’ functions, such as neutrophil migration and activation of B-cells and T-cells [11].

Heat shock proteins (HSPs) are molecular chaperones that assist in the folding and refolding of proteins and prevent protein aggregation. HSPs are upregulated in response to the acute exercise, contributing to the cellular adaptive response followed by a downregulation in the period of recovery [6,51,52,53]. As molecular chaperones, they assist in protein repair, reducing the impact of mechanical stress and oxidative damage arising from exercise. They also modulate both innate and adaptive immune responses, helping athletes maintain immune function during periods of intense training [54]. Glutamine has been shown to enhance HSP expression both in vivo and in vitro by inducing trans-activation and expression of heat shock factor 1 (HSF1) [8,55]. The activation might involve O-GlcNAcylation of the HSF1 molecule, thereby influencing its stability [9,49].

### 3.5. Theoretical Considerations of Glutamine Supplementation in Exercising Individuals

Exercise causes micro-trauma in muscle tissue, increases oxidative stress, and induces inflammatory responses [2,47]. Muscle recovery after exercise is crucial for adaptation to training and injury prevention. Skeletal muscle adaptation involves complex biochemical and molecular processes that regulate energy metabolism, intracellular signaling, and gene expression [31,32]. Glutamine levels in plasma fluctuate with exercise intensity and duration and influence immunologic and metabolic responses to exercise [4,8,20,33]. Glutamine is a common denominator of metabolic pathways involved in redox homeostasis, energy sensing, anabolic signaling, intestinal barrier integrity, and immune cell function, making it a potential modulator of muscle adaptation, performance, and recovery under conditions of exercise-induced stress.

**Figure 2:**
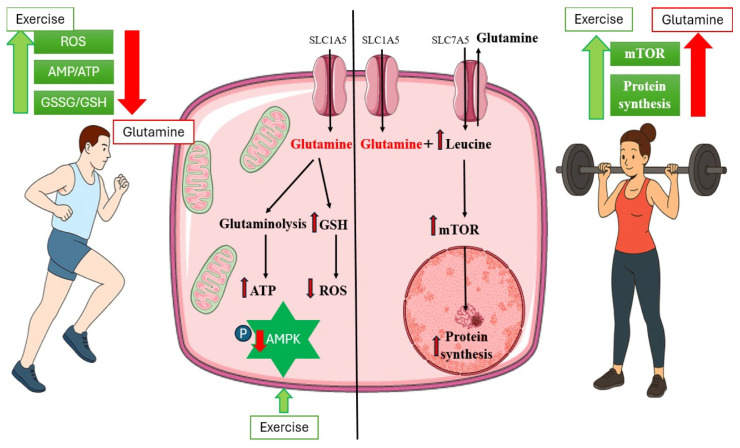
Effects of glutamine on the molecular mechanisms involved in skeletal muscle adaptation to exercise. Exercise, especially endurance or prolonged exercise, increases ROS, the AMP/ATP ratio, and the GSSG/GSH ratio, leading to activation of AMPK. Glutamine supports the TCA cycle, GSH production, and ROS neutralization, thereby affecting AMPK activity. Resistance exercise activates mTOR signaling and protein synthesis. Glutamine potentiates this effect by modulating leucine uptake via solute carrier transporters SLC1A5 and SLC7A5. Green arrow—exercise effects; Red arrow—glutamine effects. Abbreviations: AMP—adenosine monophosphate; AMPK—AMP-activated protein kinase; ATP—adenosine triphosphate; GSH—glutathione; GSSG—glutathione disulfide; mTOR—mammalian target of rapamycin; TCA—tricarboxylic acid; P—phosphorylation; ROS—reactive oxygen species; SLC1A5—Solute Carrier Family 1 Member 5; SLC7A5—Solute Carrier Family 7 Member 5.

In the skeletal muscles, a significant role in nutrient-sensing and metabolic regulation belongs to protein kinases, AMP-activated protein kinase (AMPK), and mTOR [56,57]. AMPK is activated in an energy-deprived state when the intracellular ratio of AMP/ATP increases to support catabolic processes and energy production, whereas mTOR is activated in nutrient-rich conditions (amino acids, glucose) and supports anabolism and growth [56]. AMPK and mTOR signaling create a negative feedback loop that prevents catabolic and anabolic processes from happening simultaneously [56]. In contracting skeletal muscle, AMPK–mTOR signaling represents an integrated response to energy charge, intracellular Ca^2+^, ROS, insulin signaling, growth factors, and amino-acid availability [56,58,59] (Figure 2).

A recent study reported that glutamine potentiates exercise-induced protein synthesis and muscle hypertrophy by activating anabolic signaling via the mTOR signaling pathway [22]. The effect of glutamine on the mTOR pathway might be mediated by the increased leucine uptake via heterodimeric antiporter by the solute carrier family 7 member 5 (SLC7A5) [39]. The synergistic effect of glutamine and leucine in activating the mTOR signaling pathway is potentiated by the fact that leucine acts as an activator of glutamine dehydrogenase (GDH) [60]. Additionally, glutamine may influence AMP-activated protein kinase (AMPK)-related pathways, which are crucial in the physiological adaptations to endurance exercise [60,61,62].

While glutamine can support anaplerosis, attenuation of AMPK activity may arise through other mechanisms not directly related to glutaminolysis, such as asparagine synthesis or GABA shunt [60,62]. Inhibition of AMPK might result in increased protein synthesis since active AMPK inhibits mTOR signaling. By contributing to glutathione synthesis, glutamine also modulates oxidative stress, which might indirectly influence AMPK signaling. As a precursor of glutathione, glutamine promotes the expression of HSPs, which stabilize proteins exposed to oxidative stress [4,6,13,48]. Both glutathione and HSPs might contribute to the limitation of exercise-induced oxidative injury in skeletal muscles. In addition, glutamine modulates key proinflammatory pathways, including nuclear factor kappa B (NF-κB) [6,8,32].

In the digestive system, glutamine supports the integrity of the intestinal mucosa, thereby preventing exercise-induced increases in intestinal permeability [3,7]. Exercise-induced muscle damage triggers local inflammation and recruitment of leukocytes that consume glutamine to sustain their function [6,8,35]. Since both leukocytes and enterocytes consume glutamine as an energy source, exercise-induced muscle damage and subsequent leukocyte recruitment might limit glutamine availability locally. Aside from reduced splanchnic perfusion leading to mucosal ischemia and enterocyte damage, factors contributing to the exercise-induced gastrointestinal syndrome might be thermal stress, oxidative stress, effects of gut microbiota, and inflammation [63,64,65]. Proposed mechanisms explaining increased intestinal permeability in relation to exercise rely on disturbances in tight-junction integrity, especially changes in phosphorylation and expression of claudins and occludins leading to paracellular leakage [3,7,66] (Figure 2).

Theoretically, pre-exercise glutamine supplementation may help maintain plasma glutamine levels, support acid–base balance, and contribute to antioxidant defense through glutathione synthesis and potential induction of heat shock protein expression. While its direct role in ATP production during exercise is limited, glutamine could indirectly influence AMPK and attenuate catabolic signaling. Enterocytes, which rely on glutamine as a major energy source, might also benefit from pre-exercise intake, supporting intestinal barrier integrity under stress. In contrast, post-exercise supplementation appears to be more relevant for recovery, as it replenishes TCA cycle intermediates (anaplerosis), facilitates muscle protein synthesis via mTOR activation, and sustains immune cell function during muscle repair and inflammation. Additionally, post-exercise glutamine availability may enhance the hexosamine biosynthetic pathway, which produces UDP-GlcNAc for O-GlcNAcylation of key signaling proteins, influencing insulin sensitivity, transcriptional regulation, and nutrient-sensing processes during recovery.

## 4. Glutamine Supplementation

### 4.1. Glutamine Supplementation in Preclinical Studies

Preclinical studies have demonstrated that glutamine supplementation, alone or in combination with alanine or leucine, exerts some beneficial effects in animal models subjected to exercise. These effects relate to the reversal of muscle damage and fatigue, inflammation, oxidative stress, and intestinal integrity, suggesting several effects relevant to muscle remodeling and recovery. However, no effect on performance was reported. In most preclinical studies, glutamine was administered in drinking water or by oral gavage. Only the study by Lu et al. [21] compared the effects of supplementation timing (pre vs. post exercise) and reported that the therapeutic effect of glutamine is more pronounced than preventive, meaning that post-exercise supplementation is more efficient in reducing muscle damage biomarkers and minimizing cardiac and kidney injury.

Rodrigues Junior et al. [22] showed that glutamine supplementation promotes anabolic signaling by modulating AMPK and mTOR signaling pathways involved in skeletal muscle protein metabolism, particularly when combined with exercise. Additionally, studies by Leite et al. [8] and Moura et al. [48] further demonstrated that glutamine increases HSPs expression in exercising animals and enhances antioxidant defenses by targeting the glutamine-glutathione axis and antioxidant enzymes such as superoxide dismutase (SOD) and glutathione peroxidase (Gpx).

In the animal model of ulcerative colitis, glutamine with or without exercise attenuated intestinal inflammation and oxidative stress, although it did not improve gastric motility [67]. Similarly, Freitas et al. [7] showed the protective role of alanyl-glutamine in maintaining intestinal barrier integrity during acute exhaustive exercise by affecting the expression of claudin-2, occludin, tight junction protein zonula occludens-1 (ZO-1), and peptide transporter 1 (PepT 1) gene.

Raizel et al. [4] reported that glutamine and alanine supplementation reduced inflammatory and muscle damage biomarkers in rats undergoing resistance training. Coqueiro et al. [35,68] explored the impact of glutamine/alanine mix or l-alanyl-l-glutamine dipeptide on central and muscle fatigue markers, showing beneficial effects on muscular damage but no effect on performance in resistance-trained rats. Interestingly, the results regarding central fatigue parameters such as serotonin and dopamine turnover depended on the supplement form (dipeptide vs. free). In a related study, the same authors demonstrated unfavorable effects such as increased adiposity, impaired plasma lipid profiles, and adipokine levels in exercising animals supplemented with glutamine and alanine [69].

Collectively, preclinical findings support the hypothesis that glutamine supplementation, alone or combined with alanine, might support exercise adaptation and recovery through modulation of oxidative stress, inflammation, and anabolic signaling. Nevertheless, the absence of performance enhancement and the limited evidence on supplementation timing (with only one study addressing this aspect) underscore the need for further research. Clinical studies are required to validate these findings and determine their relevance to human athletes. Table 1 summarizes data from preclinical studies.

### 4.2. Glutamine Supplementation in Clinical Studies

While preclinical studies provide mechanistic insights and suggest potential benefits, clinical trials offer a more direct evaluation of glutamine effects in exercising individuals. Most studies were randomized controlled trials (RCTs) employing parallel or crossover methodology, but they varied significantly in the number of participants. Additionally, the studies included different populations such as healthy adults, professional athletes, elderly individuals, and adolescents with type 1 diabetes. The doses varied from 0.15 to 0.9 g/kg/day or between 0.6 and 20 g/day, with some studies combining glutamine with carbohydrates, maltodextrin, probiotics, alkaline water, or other amino acids. Based on the duration of glutamine supplementation, the studies were categorized as single dose, acute (up to 14 days), or chronic administration (15 days or more). Additionally, based on the glutamine supplementation timing, the studies were categorized as pre-exercise or post-exercise supplementation. Some studies employed combined protocol administration (both pre- and post-exercise).

#### 4.2.1. Pre-Exercise Glutamine Supplementation in Clinical Trials

Glutamine was supplemented pre-exercise in 11 studies (Table 2). Four studies reported effects regarding intestinal permeability with conflicting results. In the study by Ogden et al. [70], a single dose of glutamine pre-exercise increased intestinal permeability (measured by lactulose/rhamnose dual sugar absorption test) in healthy adults but had no effect on intestinal fatty acid-binding protein (I-FABP) or microbial translocation during exhaustive running at 40 °C. In trained cyclists, a single dose of glutamine before a 20 km time trial had no effect on performance tests, endotoxin translocation, I-FABP, interleukin 6 (IL-6), and tumor necrosis factor α (TNF-α) [71]. Although this study did not directly determine intestinal permeability, there was no change observed in I-FABP plasma levels in glutamine-supplemented cyclists, showing no effect on intestinal epithelial injury.

In contrast, Pugh et al. [25] showed a dose-dependent decrease in intestinal permeability (measured by lactulose/rhamnose dual sugar absorption test) and I-FABP, reflecting a decrease in intestinal injury, after a single dose of glutamine pre-exercise given to healthy males running at 30 °C. A decrease in intestinal permeability (measured by plasma lactulose to mannitol ratio) and I-FABP levels was also observed as an effect of 6-day combined glutamine and cystine supplementation with a single dose given pre-exercise in young males [72].

Additionally, three studies with pre-exercise design reported effects on the immune system, cytokine production, and T-cell response. Acute glutamine supplementation has an insignificant effect on salivary cytokines (IL-6, IL-10, TNF-α) and IgA but decreases cytokine production in monocytes (IL-1β, TNF-α) except for IL-6 [24,27]. In addition, a single dose of glutamine increases absolute T cell count (CD3+) and the count of CD8+ T cells but produces no effect on NK/neutrophils, CD4+/CD8+ ratio, or CD19+ lymphocytes [73].

Two studies reported effects on oxidative stress and muscle damage. In healthy adults, pre-exercise glutamine supplementation for 14 days was able to restore glutathione levels, reduce inflammation and oxidative stress biomarkers, and improve total anti-oxidative capacity [26]. Additionally, pre-exercise administration of glutamine for 20 days reduced muscle enzymes (creatine kinase, lactate dehydrogenase) and IL-6 levels but did not improve performance test results (vertical jump, agility T-test, and 20-m sprint) in professional athletes [28]. However, acute supplementation with glutamine reduced the rate of perceived exertion (RPE) during intense exercise in simulated hypoxia [74].

Pre-exercise glutamine might be effective in protecting intestinal integrity, modulating immune responses, reducing oxidative stress, and muscle damage. However, the number of studies reporting the effects of pre-exercise glutamine supplementation is relatively small, and their results remain conflicting. Several trials demonstrated reductions in exercise-induced intestinal permeability and inflammatory markers, especially under stress conditions like hypoxia, which might be crucial for athletes who train intensely under unfavorable conditions. The number of studies reporting effects on athletic performance is small, although some results appear promising.

#### 4.2.2. Post-Exercise Glutamine Supplementation in Clinical Trials

Only two trials reported effects of post-exercise glutamine supplementation (Table 3). A single dose of glutamine and alanine administered post-exercise restored reduced citrulline and I-FABP levels after PA in healthy young men, reflecting improvement in intestinal mucosa integrity [75]. Additionally, three weeks of glutamine supplementation after intensive training enhanced oral mucosa immunity, improved hormonal status (testosterone to cortisol ratio), and reduced upper respiratory tract infection incidence in combat-sport athletes [76]. Those recent findings suggest that post-exercise glutamine supplementation might be effective in enhancing mucosal integrity, immune function, and the prevention of overtraining syndrome. Although studies showed promising results, more evidence is needed.

#### 4.2.3. Combined Pre- and Post-Exercise Glutamine Supplementation in Clinical Trials

Mixed protocol combining pre- and post-exercise supplementation in healthy individuals who exercise was reported in one study (Table 4). Legault et al. reported an increase in muscle strength assessed through relative peak torque and a decrease in muscle soreness ratings post-exercise in healthy adults [23].

#### 4.2.4. Clinical Trials with Non-Specified Timing of Glutamine Supplementation

Several trials did not specify supplementation timing in healthy exercising individuals (Table 5). The studies with no reported glutamine supplementation timing lasted from 7 days to 3 months. Glutamine supplementation also reduced oxidative stress markers and high-sensitivity C-reactive protein (hs-CRP) in healthy young males after exhaustive exercise, suggesting its potential benefit for cardiovascular health and recovery [77]. Boxing athletes benefited in post-exercise recovery from concurrent ingestion of alkaline water and glutamine, which enhanced salivary α-amylase activity and testosterone concentration [78]. In contrast, chronic combined probiotic and glutamine intake in ultra-endurance athletes had no effect on HSP 72 expression and no significant effect on performance during a multi-day exercise event [79].

#### 4.2.5. Clinical Trials Involving Specific Populations

Five trials involved specific populations such as elderly or elderly women, adult women with HIV/AIDS, or adolescents with type 1 diabetes (Table 6). Those trials did not specify supplementation timing but still offer valuable insights, particularly in specific populations in relation to exercise. The studies involving specific populations on glutamine supplementation lasted from 7 days to 3 months.

In the elderly, glutamine supplementation enhanced the anti-inflammatory and antioxidant effects of exercise, suggesting a synergistic benefit for aging populations [80,81]. A similar synergistic effect of glutamine supplementation and exercise was observed in elderly women on the improvement of muscle performance, glycemic control, and oxidative stress markers [82]. In addition, glutamine supplementation in the elderly increased immunoglobulin synthesis and CD4+ T cell production post vaccination [83].

In adolescents with type 1 diabetes, resistance exercise combined with glutamine supplementation led to a reduction in body fat mass, though it had no effect on lean body mass, glycated hemoglobin (HbA1c), or daily insulin dose [84]. Similarly, another study including adolescents with type 1 diabetes, with a combined pre- and post-exercise supplementation design, demonstrated a reduction in blood glucose levels and increased incidence of nocturnal hypoglycemia [85].

In women with HIV/AIDS, short-term glutamine dipeptide supplementation partially reduced the increase in TNF-α post-exercise and enhanced cognitive performance after resistance exercise [86]. Regardless of supplementation timing, glutamine seems to produce some health benefits in both young and elderly people involved in PA, as well as athletes, and certain clinical conditions such as HIV/AIDS.

## 5. Discussion

In this narrative review, we synthesized the biochemical and physiological mechanisms underlying the relevance of glutamine to exercise and evaluated preclinical and clinical evidence on glutamine supplementation outcomes with a particular focus on timing of administration. While most studies found in the literature involved healthy, exercising individuals, we additionally included trials conducted in specific clinical or age-related populations when these provided relevant biochemical evidence regarding the effects of glutamine in exercising individuals.

Preclinical studies show that glutamine might modulate mTOR-linked anabolism, AMPK signaling, antioxidant defense (glutathione, HSPs), intestinal barrier integrity, and immune function during exercise, as shown in Table 1. The major effects appear to be reduced tissue injury and inflammation; however, there was no effect on performance, and one study reported unfavorable effects on lipid metabolism [69]. Timing has not been widely studied, with one study favoring post-exercise dosing for therapeutic effects [21]. Human trials report mitigation of immune and intestinal disruption caused by exercise, oxidative stress, and inflammation reduction, yet fail to consistently demonstrate effects on muscle strength, performance, and recovery, as shown in Table 2, Table 3, Table 4, Table 5 and Table 6. Results across studies are mixed, and the insufficient evidence limits the ability to define practice-oriented recommendations regarding glutamine supplementation in exercising individuals and an optimal timing strategy. This is at least in part a reflection of the heterogeneity of included studies.

In athletes training in the heat or during strenuous endurance sessions, acute pre-exercise glutamine can attenuate intestinal permeability and reduce intestinal epithelium damage biomarkers (I-FABP), although effects are inconsistent across protocols [20,70,71,72]. Additionally, pre-exercise glutamine could modulate immune function, support antioxidant defenses, and reduce tissue-injury biomarkers, thereby supporting recovery-focused outcomes [21,22,23,73]. Evidence for post-exercise supplementation, although limited, suggests potential benefits for hormonal regulation that may aid in preventing overtraining syndrome, as well as reductions in the incidence of upper respiratory tract infections among athletes undergoing intense training [76]. Across studies employing either pre-, post-, or combined timing strategy, glutamine supplementation did not consistently improve performance outcomes [18,23,71].

The doses varied from 0.15 to 0.9 g/kg/day or between 0.6 and 20 g/day, with several trials also using dipeptide forms. Due to high variability in dosing regimens, no optimal dose can be recommended, and any use should be individualized to the athlete’s specific needs, environmental conditions, and tolerance. Safety data indicate that, although glutamine is well-tolerated at moderate intakes, higher doses can elicit dose-dependent gastrointestinal discomfort [87]. Risk-assessment analyses using the Observed Safe Level approach identify 14 g/day as the upper safe chronic intake level for healthy adults, even though some studies have administered higher amounts without documented adverse events [88].

Acute trials in healthy, physically active men demonstrate that ingestion of 0.9 g/kg fat free mass (FFM) produces greater, but still mild gastrointestinal symptoms, such as nausea, upper gastrointestinal pain, and discomfort, compared with lower doses [87]. Intakes between 0.3–0.6 g/kg are well tolerated [87]. These safety considerations are particularly relevant in athletes, where even minor gastrointestinal symptoms can impair pacing and overall performance, and may, in some cases, lead to discontinuation of training or competition. Consequently, more conservative dosing, especially when glutamine is consumed before exercise, is advisable to minimize the likelihood of exercise-limiting GI discomfort while still allowing for potential physiological benefits.

Previous systematic review by Ramezani Ahmadi et al. [29] synthesized data on glutamine supplementation in athletes from 47 studies (25 included in the quantitative meta-analysis) published up to January 2017. Their findings suggest that while glutamine does not improve performance or immune function, it may influence metabolic recovery and neutrophil counts depending on dosage and formulation. Another systematic review with meta-analysis by Abbasi et al. [89], which included 10 studies from 1998 to 2014, further suggested that glutamine doses above 30 mg/day, administered for less than two weeks, may significantly reduce intestinal permeability. The timing of administration and its potential effect on specific outcomes were not assessed.

From a biochemical standpoint, glutamine is positioned at the intersection of anaplerosis, antioxidant defense, and nutrient signaling. First, glutaminolysis replenishes the TCA cycle and supports bioenergetics in rapidly dividing and stressed cells, while generating ammonia for acid–base balance regulation [10,32,40,41]. Second, glutamine supplies glutamate for GSH synthesis and indirectly sustains GSH recycling through NADPH-dependent glutathione reductase [13,14]. Third, the HBP generates UDP-GlcNAc, enabling O-GlcNAcylation of various proteins (including HSF1 and components upstream of mTOR/AMPK), with implications for insulin signaling, transcription, and protein synthesis [11,12,49,50]. Fourth, intracellular glutamine facilitates SLC7A5-mediated leucine influx, thereby potentiating mTOR activation and protein synthesis [39,56,57].

These pathways might translate into timing hypotheses. Pre-exercise glutamine administration may prevent the decrease in circulating glutamine during strenuous exercise, support enterocyte energetics to mitigate exercise-induced gastrointestinal syndrome, and prime antioxidant defenses and HSP responses to reduce acute oxidative and inflammatory damage. Post-exercise glutamine supplementation may support anaplerosis and the TCA cycle, mTOR-driven protein synthesis, and immune cell function during tissue repair. The timing of glutamine supplementation should be interpreted cautiously. Since the number of studies directly comparing pre- versus post-exercise administration protocols is extremely limited, available data do not permit practice recommendations beyond hypothesis-generating statements.

Even though most of the studies were RCTs, there was heterogeneity in glutamine supplementation dosing, formulation, timing, and duration, which limits the strength of available conclusions. Several studies used glutamine in dipeptide form or in combination with other substances such as maltodextrin or other amino acids (alanine and cysteine). Additionally, while some studies focused on professional athletes, the majority involved recreational or untrained individuals, even specific populations such as the elderly or people with a clinical condition. A significant limitation across studies is the small sample size of the included trials. Moreover, most clinical trials enrolled male participants, potentially masking sex-specific responses.

Future work should prioritize larger RCTs that include both recreational and professional athletes, with balanced participation from both women and men. To reduce inconsistencies seen across studies, investigators should use standardized exercise and supplementation protocols, clearly specifying dose, timing, formulation (free vs. dipeptide), and duration, and then replicate promising findings to confirm reliability. In parallel, targeted preclinical studies are needed to clarify how glutamine affects key molecular pathways underlying its observed effects. Finally, direct comparative trials are essential to determine whether timing meaningfully alters physiological, biochemical, or clinical outcomes. Ideally, these studies should integrate biomarkers with performance measures and athlete-reported symptoms, while accounting for environmental stressors such as heat or hypoxia, to identify the athlete subgroups most likely to benefit from targeted, context-specific glutamine strategies.

## 6. Conclusions

Despite the widespread use of glutamine among athletes, scientific evidence supporting its efficacy is still limited. Both preclinical and clinical studies reported potential benefits on intestinal integrity, oxidative stress, muscle damage biomarkers, immune function, and inflammation, yet findings are inconsistent. The timing of supplementation might influence outcomes; however, the small number of studies, heterogeneity, and conflicting results make it difficult to draw practice-oriented recommendations. Overall, glutamine appears plausible as a supportive nutrient during exercise across various populations, including athletes and individuals with clinical conditions.

At present, routine use for performance appears not to be justified. However, targeted use may be considered in the prevention of exercise-induced gastrointestinal syndrome, especially in athletes training in the heat or during strenuous endurance exercise sessions, or in supporting post-exercise recovery. More conservative dosing is advisable, particularly when glutamine is consumed before exercise, to reduce the risk of gastrointestinal discomfort that could potentially compromise or limit performance. Overall, there is a need for well-designed, adequately powered preclinical and clinical trials—especially those using standardized dosing protocols, directly comparing timing strategies, and including female participants to better define under what conditions glutamine supplementation is most beneficial.

## Figures and Tables

**Table 1:** Summary of preclinical studies investigating glutamine supplementation effects on physiological and biochemical outcomes in animal models.

Author	Animals	Supplementation	Exercise Protocols	Main Findings
Raizel et al. [4]	Wistar rats, 2 months, male	Ala, Gln+Ala or DIP (4% in water for 21 days, ad libitum)	Resistance training (ladder-climbing protocol with extra weights tied to their tails, 8 weeks)	↑ Gln, ↓ reduced inflammation (↓ TNF-α, ↓ IL-1β, ↑ IL-6, ↑ IL-10, ↑ MCP-1, ↓ NF-κB), ↓ muscle damage (↓ CK, ↓ LDH), ↑ HSP70
Freitas et al. [7]	Wistar rats, age NR, male	DIP, 1.5 g/kg, 6 weeks, post-exercise	Swimming (12 weeks) + acute swimming exhaustive test after training	↓ Intestinal permeability (↓ urinary lactulose and mannitol levels), improved barrier function, (↓ claudin-2, occludin, ZO-1, PepT 1 gene expression)
Leite et al. [8]	Wistar rats, 8 weeks, male	Ala, Gln+Ala or DIP (4% in water for 21 days, ad libitum)	Resistance training (ladder-climbing protocol with extra weights tied to their tails, 8 weeks)	↑ Gln, ↓oxidative stress (↓ GSSG/GSH, ↓ TBARS), ↓ muscle damage (↓ CK), ↑ HSP synthesis (↑ HSF-1, ↑ HSP-27)
Lu et al. [21]	Sprague–Dawley rats, age and sex NR	Gln, 1 g/kg, pre or post exercise, in drinking water	Exhaustive exercise (treadmill with increments to a maximum running speed of 15 miles/min with no incline)	Treatment group: ↓ CK-MM, ↑ red blood cell count and platelet count, ↓ cardiac and kidney damage. The therapeutic effect of Gln was more effective than preventive.
Rodrigues Junior et al. [22]	Wistar rats, age NR, male	Gln, 1 g/kg/day, 5 weeks, oral gavage	Resistance training (ladder-climbing protocol with extra weights tied to their tails, 5 weeks)	Gln + exercise group: ↑ protein synthesis signaling (AKT/mTOR)
Coqueiro et al. [35]	Wistar rats, 60 days, male	Ala, Gln+Ala or DIP (4% in water for 21 days, ad libitum)	Resistance training (ladder-climbing protocol with extra weights tied to their tails, 8 weeks)	↓ Muscle fatigue, ↓ muscle damage (↓ LDH, ↓ CK), ↑ Gln, ↑ Glu
Moura et al. [48]	Wistar rats, 7 weeks, male	Gln, 0.75 g/kg, single dose, oral gavage, postexercise	Acute exercise stress model (treadmill without inclination at the speed of 18 m/min for one hour)	↑ HSP-60 expression, ↑ Gpx, ↔ SOD, ↔ HSP 25, HSP 70, HSP 90 expression
de Oliveira Santos et al. [67]	Wistar rats, age NR, male	Gln, 1 g/kg/day, 8 weeks, oral gavage	Strength (jump training 8 weeks with progressive overload) or/and endurance exercise (swimming without overload for 8 weeks)	↓ Inflammation (↓ IL-1β, ↓ IL-6, ↓ TNF-α), ↓ oxidative stress (↓ MDA, ↓ SOD, ↓ myeloperoxidase, ↓ NO-2/NO-3)
Coqueiro et al. [68]	Wistar rats, 2 months, male	Ala, Gln+Ala or DIP (4% in water for 21 days, ad libitum)	Resistance training (ladder-climbing protocol with extra weights tied to their tails, 8 weeks)	Central fatigue markers (hypothalamic 5-HT, 5-HT/DA ratio): ↑ DIP, ↓ G+A, ↔ performance (MCC test)
Coqueiro et al. [69]	Wistar rats, 2 months, male	Ala, Gln+Ala or DIP (4% in water for 21 days, ad libitum)	Resistance training (ladder-climbing protocol with extra weights tied to their tails, 8 weeks)	↑ Adiposity, impaired lipid profile (↑ LDL-c, ↑ TC), ↑ IL-6, ↑ IL-10

↓—significantly decreased; ↑—significantly increased; ↔—no change in concentration or effect. Abbreviations: 5-HT—serotonin; T AKT/mTOR—AKT (Protein kinase B)/mammalian target of rapamycin; Ala—Alanine; CK—Creatine Kinase; CRP—C-reactive Protein; DA—Dopamine; DIP—Dipeptide (alanyl-glutamine); FFA—Free Fatty Acids; GPx—Glutathione Peroxidase; GSH—Glutathione; GSSG—Glutathione Disulfide; Gln—Glutamine; HDL-c—High-Density Lipoprotein Cholesterol; HSF-1—Heat Shock Factor 1; HSP—Heat Shock Protein; hs-CRP—High-Sensitivity C-Reactive Protein; Ig—Immunoglobulin; IL—Interleukin; LDH—Lactate Dehydrogenase; MCC Test—Maximum Carrying Capacity Test; MCP-1—Monocyte chemoattractant protein-1; MDA—Malondialdehyde; MMP—Matrix Metalloproteinase; NF-κB—Nuclear factor kappa-light-chain-enhancer of activated B cells; NO—Nitric Oxide NO-2/NO-3—Nitrite and Nitrate; NR—Not reported; ox-LDL—Oxidized Low-Density Lipoprotein; PepT-1—Peptide transporter 1 (solute carrier family 15 member 1-SLC15A1); PON-1—Paraoxonase-1; PRx—Peroxidase; RPE—Rating of Perceived Exertion; SOD—Superoxide Dismutase; TAC—Total Antioxidant Capacity; TBARS—Thiobarbituric Acid-Reactive Substance; TC—Total Cholesterol; TNF-α—Tumor Necrosis Factor Alpha; TP—Total Protein; ZO-1—Zonula Occludens-1.

**Table 2:** Summary of randomized controlled trials (RCTs) investigating glutamine supplementation in relation to exercise with pre-exercise glutamine administration.

Author	Study Design	Subjects	Exercise Protocol	Supplementation	Major Effects
Caris et al. [24]	RCT, double-blind	Healthy adults (n = 15), male	Strenuous exercise at 70% VO_2_peak	20 g/day Gln for 6 days + 200 mL 8% MD on the test day	↔ Inflammation (salivary IgA, IL-6, IL-10, TNF-α)
Pugh et al. [25]	RCT, placebo-controlled, crossover	Healthy adults, recreationally active (n = 10), male	60-min treadmill run at 70% VO_2_max, (at 30 °C, humidity 40–45%)	0.25 g/kg, 0.5 g/kg, and 0.9 g/kg Gln, single dose	↓ Intestinal permeability (L/R test), ↓ I-FABP
Nemati et al. [26]	RCT, placebo-controlled	Healthy young adults (n = 30), male	Exhaustive exercise (Bruce protocol until experiencing fatigue)	0.3 g/kg/day Gln + sugar, 14 days	↑ Gln, ↓ oxidative stress (↓ MDA,↑ TAC) ↓ hs-CRP, ↔ MMP-2, ↔ MMP-9
Caris et al. [27]	RCT, crossover, double-blind pilot study	Healthy adults, physically active (n = 15), male	Exhaustive exercise under simulated hypoxia (70% VO2max)	20 g/day Gln for 6 days + 200 mL 8% MD on the test day	↑ Gln, ↓ inflammation (↓ IL-1β, ↓ TNF-α, ↔IL-6), ↔ erythropoietin, ↔ myeloperoxidase
Córdova-Martínez et al. [28]	RCT, double blind, placebo-controlled, crossover	Professional basketball players (n = 12), male	Players followed their regular training and competition routines.	6 g/day Gln for 20 days	↓ Muscle damage (↓ CK, ↓ LDH), ↓ inflammation (↓ IL-6, ↓ CRP), ↔ performance tests (vertical jump, agility T-test, and 20-m sprint)
Ogden et al. [70]	RCT, placebo-controlled, crossover	Healthy adults (n = 14), male	Running in the heat (40 °C and 40% humidity)	0.3 g/kg Gln, single dose	↑ Intestinal permeability (L/R test), ↔ I-FABP, ↔ Bacteroides/total 16S DNA ratio
Osborne et al. [71]	RCT, placebo-controlled	Trained cyclists (n = 12), male	Two 20-km time trials (35 °C, 50% humidity)	0.9 g/kg FFM, single dose	↔ Endotoxin translocation, ↔ I-FABP, ↔ inflammation (↔ IL-6, ↔ TNF-α),↔ performance tests
Tataka et al. [72]	RCT, crossover	Young adults (n = 16), male	1-h run at 75% VO_2_max	0.2 g Cys + 1.0 g Gln + 1.2 g MD, 3×/day for 5 days + single dose before exercise on day 6	↓ Intestinal permeability (L/M ratio), ↓ I-FABP
Zheng et al. [73]	RCT, crossover	Healthy, untrained adults (n = 13), male	Treadmill running at 40% VO_2_max to exhaustion (38 °C, 60% humidity).	0.6 g/kg Gln, single dose	↑ CD3+ and CD3+ CD8+ T cells; ↔ NK/neutrophils, ↔ CD4+/CD8+ ratio, ↔ CD19+ lymphocytes
Caris et al. [74]	RCT, double-blind, crossover	Healthy adults (n = 9), male	Running at 70% VO_2_peak under simulated hypoxia	20 g/day Gln for 6 days + 200 mL 8% MD on the test day	↓ RPE, ↑ glycemia, ↔ heart rate, ↔ lactate

↓—significantly decreased; ↑—significantly increased; ↔—no change in concentration or effect. Abbreviations: CD—Cluster of Differentiation: CK—Creatine Kinase; CRP—C-reactive Protein; Cys—Cysteine; Gln—Glutamine; hs-CRP—High-Sensitivity C-Reactive Protein; I-FABP—Intestinal Fatty Acid-Binding Protein; Ig—Immunoglobulin; IL—Interleukin; FFM—Fat Free Mass; LDH—Lactate Dehydrogenase; L/M ratio—lactulose to mannitol ratio; L/R test—Lactulose/rhamnose absorption test; MDA—Malondialdehyde; MD—Maltodextrin; MMP—Matrix Metalloproteinase; NK—Natural Killer T cells; RCT—Randomized Controlled Trial; RPE—Rating of Perceived Exertion; TAC—Total Antioxidant Capacity; TNF-α—Tumor Necrosis Factor Alpha; VO_2_max—Maximal Oxygen Uptake.

**Table 3:** Summary of randomized controlled trials (RCTs) investigating glutamine supplementation in relation to exercise with post-exercise glutamine administration.

Author	Study Design	Subjects	Exercise Protocol	Supplementation	Major Effects
Kartaram et al. [75]	RCT, crossover	Healthy recreational-active cyclists (n = 15), male	A rest and four-cycle ergometer protocols under varying intensity and hydration	7.5 g Gln + 7.5 g Ala, single dose	↑ Plasma citrulline, ↑ I-FABP
Lu et al. [76]	RCT, double-blind, placebo-controlled	Combat-sport athletes (n = 21), male	2-h session each day 5 days a week, with an intensity 80% of their maximum heart rate	0.3 g/kg/day Gln, 3 weeks	↑ Salivary IgA; ↑ Salivary NO; ↑ Testosterone/Cortisol ratio; ↓ Upper Respiratory Tract Infection; ↑ well-being scores

↓—significantly decreased; ↑—significantly increased. Abbreviations: Ala—Alanine; Gln—Glutamine; I-FABP—Intestinal Fatty Acid-Binding Protein; Ig—Immunoglobulin; NO—Nitric Oxide; RCT—Randomized Controlled Trial.

**Table 4:** Summary of randomized controlled trials (RCTs) investigating glutamine supplementation in relation to exercise with combined glutamine administration.

Author	Study Design	Subjects	Exercise Protocol	Supplementation	Major Effects
Legault et al. [23]	RCT, double-blind, placebo-controlled crossover	Healthy young adults (n = 16), male and female	Unilateral knee extension at 125% of maximum concentric force, 8 sets of 10 repetitions with 2-min rest intervals	0.3 Gln g/kg/day + 0.3 g/kg/day MD; Day 1—pre- and post-exercise. Day 2 and 3—one dose before testing	↑ Peak torque at 30°/s post-exercise. ↓ Muscle soreness. Better response in men.

↓—significantly decreased; ↑—significantly increased. Abbreviations: MD—Maltodextrin; RCT—Randomized Controlled Trial.

**Table 5:** Summary of randomized controlled trials (RCTs) investigating glutamine supplementation in relation to exercise with non-specified timing of glutamine supplementation.

Author	Study Design	Subjects	Exercise Protocol	Supplementation	Major Effects
Lu et al. [76]	RCT, crossover	Boxing athletes (n = 9), male	Regular 2 h boxing training	0.15 g/kg Gln + 400 mL alkaline water for 3 weeks	↑ α-Amylase activity/TP, ↑ salivary testosterone; ↔ lactoferrin/TP, ↔ IgA/TP, ↔ cortisol
Alipanah-Moghadam et al. [77]	RCT	Healthy young adults (n = 30), male	Exhaustive exercise	0.3 g/kg/day Gln + 25 g sugar for 14 days	↓ Leptin, ↓ Cholesterol, ↓ ox-LDL, ↓ ox-LDL/HDL ratio, ↓ IL-6
Marshall et al. [79]	RCT	Ultra-endurance athletes (n = 32)	7-day ultra-marathon	Probiotics + Gln (5 g/day) for 12 weeks	↔ Hsp72 expression. Probiotics + Gln group: 9% faster than control

↓—significantly decreased; ↑—significantly increased; ↔—no change in concentration or effect. Abbreviations: CET—Combined-Exercise Training; CD—Cluster of Differentiation: GPx—Glutathione Peroxidase; GSH—Glutathione; GSSG—Glutathione Disulfide; Gln—Glutamine; HDL-c—High-Density Lipoprotein Cholesterol; Ig—Immunoglobulin; IL—Interleukin; NO—Nitric Oxide; NP—Non-Practitioners; ox-LDL—Oxidized Low-Density Lipoprotein; PON-1—Paraoxonase-1; PRx—Peroxidase; RCT—Randomized Controlled Trial; TAC—Total Antioxidant Capacity; TP—Total Protein.

**Table 6:** Summary of randomized controlled trials (RCTs) investigating glutamine supplementation in specific populations.

Author	Study Design	Subjects	Exercise Protocol	Supplementation	Major Effects
Almeida et al. [80]	RCT, placebo-controlled	Elderly individuals (n = 83), male and female	Moderate-intensity CET vs. NP	0.3 g/kg/day Gln for 30 days	↓ Salivary NO, ↑ salivary uric acid, ↓ salivary GSH, ↑ salivary PRx activity, ↔ albumin, ↔ TAC
Pires et al. [81]	RCT, double blind, placebo-controlled	Elderly individuals (n = 83), male and female	Moderate-intensity CET vs. NP	0.3 g/kg/day Gln for 30 days	↑ HDL-c, ↑ PRx, ↑ GPx, ↑ PON-1 in CET group
Amirato et al. [82]	RCT, placebo-controlled	Elderly women (n = 44)	Sedentary or engaged in regular PA (≥24 months)	10 g Gln + 10 g MD for 30 days	↑ Knee extensor/flexor muscle average power, ↓ D-fructosamine, ↓ insulin levels, ↓ oxidative stress (↓ TBARS, ↑ GSH/GSSG)
Monteiro et al. [83]	RCT, placebo-controlled	Elderly individuals (n = 84), male and female	Moderate-intensity CET vs. NP	0.3 g/kg/day Gln for 30 days	↑ IgA, ↑ IgM, ↑ naive and effector CD4+ T cells
Hasan et al. [84]	Feasibility study with randomized groups (glutamine vs. placebo)	Adolescents with type 1 diabetes, sedentary (n = 14)	6 × 1-min resistance-based activities, performed 3 times daily	0.5 g/kg/day Gln for 3 months	↓ Body fat mass. ↔ lean body mass, ↔ HbA1c, ↔ daily insulin dose
Torres-Santiago et al. [85]	RCT, double-blind, crossover	Adolescents with type 1 diabetes (n = 13), male and female	Four 15-min treadmill sessions with 5-min rest intervals	0.25 g/kg, single dose pre and post exercise	↓ Blood glucose, ↑ nocturnal hypoglycemia frequency, ↔ insulin sensitivity
de Souza et al. [86]	RCT, double-blind, crossover	Women with HIV/AIDS (n = 10)	Acute resistance training session	20 g/day Gln dipeptide, 7 days	Improved cognitive recovery

↓—significantly decreased; ↑—significantly increased; ↔—no change in concentration or effect. Abbreviations: CET—Combined-Exercise Training; CD—Cluster of Differentiation: GPx—Glutathione Peroxidase; GSH—Glutathione; GSSG—Glutathione Disulfide; Gln—Glutamine; HbA1c—glycated hemoglobin (hemoglobin A1c); HDL-c—High-Density Lipoprotein Cholesterol; HIV/AIDS—Human Immunodeficiency Virus/Acquired Immunodeficiency Syndrome; Ig—Immunoglobulin; IL—Interleukin; NO—Nitric Oxide; NP—Non-Practitioners; ox-LDL—Oxidized Low-Density Lipoprotein; PON-1—Paraoxonase-1; PRx—Peroxidase; RCT—Randomized Controlled Trial; TAC—Total Antioxidant Capacity; TBARS—Thiobarbituric Acid Reactive Substances; TBARS—Thiobarbituric Acid Reactive Substances; TP—Total Protein.

## Data Availability

No new data were created or analyzed in this study. Data sharing is not applicable to this article.

## Abbreviations

The following abbreviations are used in this manuscript:

AKT	AKT kinase (protein kinase B)
Ala	Alanine
AMP	Adenosine Monophosphate
AMPK	AMP dependent kinase
ATP	Adenosine Triphosphate
ATs	Aminotransferases
CD	Cluster of Differentiation
CET	Combined-Exercise Training
CK	Creatine Kinase
CL	Citrate Lyase
CoA	Coenzyme A
Cys	Cysteine
FFA	Free Fatty Acids
GABA	γ–Aminobutyric Acid
GDH	Glutamate Dehydrogenase
GLS	Glutaminase
Gln	Glutamine
Glu	Glutamate
GlcNAc	N–Acetyl Glucosamine
GLUT	Glucose Transporter
GPx	Glutathione Peroxidase
GR	Glutathione Reductase
GSH	Glutathione
GSSG	Oxidized Glutathione
HIV/AIDS	Human Immunodeficiency Virus/Acquired Immunodeficiency Syndrome
HDL-c	High-Density Lipoprotein Cholesterol
HSF1	Heat Shock Factor 1
HSP	Heat Shock Proteins
hs-CRP	High-Sensitivity C-Reactive Protein
I-FABP	Intestinal FattyAcid Binding Protein
Ig	Immunoglobulin
IL	Interleukin
FFM	Fat Free Mass
LDH	Lactate Dehydrogenase
MD	Maltodextrin
MDH	Malate Dehydrogenase
ME	Malic Enzyme
MMP	Matrix Metalloproteinase
mTOR	Mammalian Target of Rapamycin
NAD	Nicotinamide Adenine Dinucleotide
NADP	Nicotinamide Adenine Dinucleotide Phosphate
NF-κB	Nuclear Factor Kappa B
NK	Natural Killer Cells
NO	Nitric Oxide
NOX2	NADPH Oxidase 2
NP	Non-Practitioners
OAA	Oxaloacetate
ox-LDL	Oxidized Low-Density Lipoprotein
PA	Physical activity
PDH	Pyruvate Dehydrogenase
PepT 1	Peptide Transporter 1
PON-1	Paraoxonase-1
PRx	Peroxidase
RCT	Randomized Controlled Trial
RPE	Rating of Perceived Exertion
SLC1A5	Solute Carrier Family 1 Member 5
SLC38A1	Solute Carrier Family 38 Member 1
SLC38A2	Solute Carrier Family 38 Member 2
SLC7A5	Solute Carrier Family 7 Member 5
SLC7A11	Solute Carrier Family 7 Member 11
SOD	Superoxide Dismutase
TAC	Total Antioxidant Capacity
TCA	Tricarboxylic Acid Cycle
TNF-α	Tumor Necrosis Factor Alpha
TP	Total Protein
URTI	Upper Respiratory Tract Infection
VO_2_max	Maximal Oxygen Uptake
ZO-1	Protein Zonula Occludens-1
2-OG	2-oxoglutarate
